# Recurrent abdominal wall mass in a hepatitis B‐positive male: An unusual case of lumbar mycetoma

**DOI:** 10.1002/ccr3.8275

**Published:** 2023-11-30

**Authors:** Alaa Tajeldeen Habeeb Abdallah, Rami Elsiddig Abdelkhalig, Elwasila Hamid, Ayman Ahmed, Emmanuel Edwar Siddig

**Affiliations:** ^1^ Rufa'a Teaching Hospital Rufaa Sudan; ^2^ General surgeon Rufa'a Teaching Hospital, Albutana University Rufaa Sudan; ^3^ Consultant Surgeon, Rufa'a Teaching Hospital Albutana University Rufaa Sudan; ^4^ Institute of Endemic diseases, University of Khartoum Khartoum Sudan; ^5^ Swiss Tropical and Public Health Institute (Swiss TPH) Allschwil Switzerland; ^6^ University of Basel Basel Switzerland; ^7^ Department of Medical Microbiology and Infectious Diseases ErasmusMC, University Medical Center Rotterdam Rotterdam the Netherlands; ^8^ Faculty of Medical Laboratory sciences University of Khartoum Khartoum Sudan

**Keywords:** abdominal wall, atypical presentation, eumycetoma, grains

## Abstract

**Key Clinical Message:**

Atypical presentations of eumycetoma can pose a challenge in the diagnosis and treatment of the disease. Healthcare providers thorough in their differential diagnosis and investigations, even in the absence of classic symptoms, in order to improve early detection and the case management for such a neglected tropical disease.

**Abstract:**

In this communication, we present a case study of an unusual presentation of eumycetoma; a fungal infection that is considered a neglected tropical disease. The patient, a 28‐year‐old male from Sudan, presented with a recurrent mass in the abdominal wall. Despite two surgeries to remove the mass, it continued to recur. Unlike typical cases of eumycetoma, this patient did not exhibit common symptoms such as painless swelling, sinuses, or grain‐containing discharge. The diagnosis was made incidentally after surgical excision of the mass. The abstract highlights the importance of recognizing uncommon presentations and maintaining a high suspicion for rare diagnoses, even in the absence of classic symptoms. Further research is needed to better understand atypical presentations of eumycetoma and improve early detection.

## 1 BACKGROUND

Mycetoma is a neglected tropical disease (NTD) that affects the skin and subcutaneous tissue.^1^ The disease is characterized by chronic granulomatous inflammation, which occurs after the causative organism is introduced into the body through skin break.^2^ Infections can be caused by either bacteria (actinomycetoma) or fungus (eumycetoma); nevertheless, the fungal disease or eumycetoma is the most prevalent worldwide.^2^ The infection is commonly manifested at the feet and hands.^3^ Eumycetoma typically presents with a classical triad of painless swelling, sinuses (abnormal openings), and discharge‐containing grains.^4^ However, due to the severe neglection of operational research in mycetoma, detection and diagnosis of mycetoma cases are challenging particularly when they are presented atypically. Unfortunately, most of mycetoma cases occur among poor communities in low‐ and middle‐income countries (LMICs), where diagnostic capacity and resources are limited that makes it more challenging to prevent, diagnose, and treat.

Mycetoma is endemic in Sudan with cases reported almost throughout the country.^2^ However, farming‐dependent communities in remote states are at higher risk of the disease. These states including Al Gaziera, Gedaref, Sennar, and White Nile report the highest prevalence of mycetoma in Sudan.^2^

In this report, we present a case of male displayed with unusual presentation of the disease. This case exhibited an atypical presentation of mycetoma that we accidentally discovered during surgical process, and we implemented further investigations to confirm.

## 2 CASE PRESENTATION

A 28‐year‐old male driver from Alhidaya, Al Gaziera State, Sudan, presented to Rufa'a Teaching Hospital on August 15th, 2023, with a recurrent left‐sided abdominal wall mass in the lumbar region. The patient first noticed a small mass in his abdomen 6 years ago, which gradually increased in size. The mass was mildly painful, primarily experienced during heavy lifting, and relieved by resting. Local anesthesia excision was performed on two occasions to remove the mass. However, 2 years after each excision, another growth of the mass recurred at the same site. The third recurrence was smaller in size than the previous two, but it displayed similar characteristics of mild pain with heavy lifting and no sinuses or discharges. The patient do not recall any history of trauma at the site. There is no family history of mycetoma, and the patient was diagnosed with hepatitis B since 2018; however, no evidence or clinical/epidemiological history about for how long they were infected with hepatitis B virus prior to the diagnosis.

**FIGURE 1:**
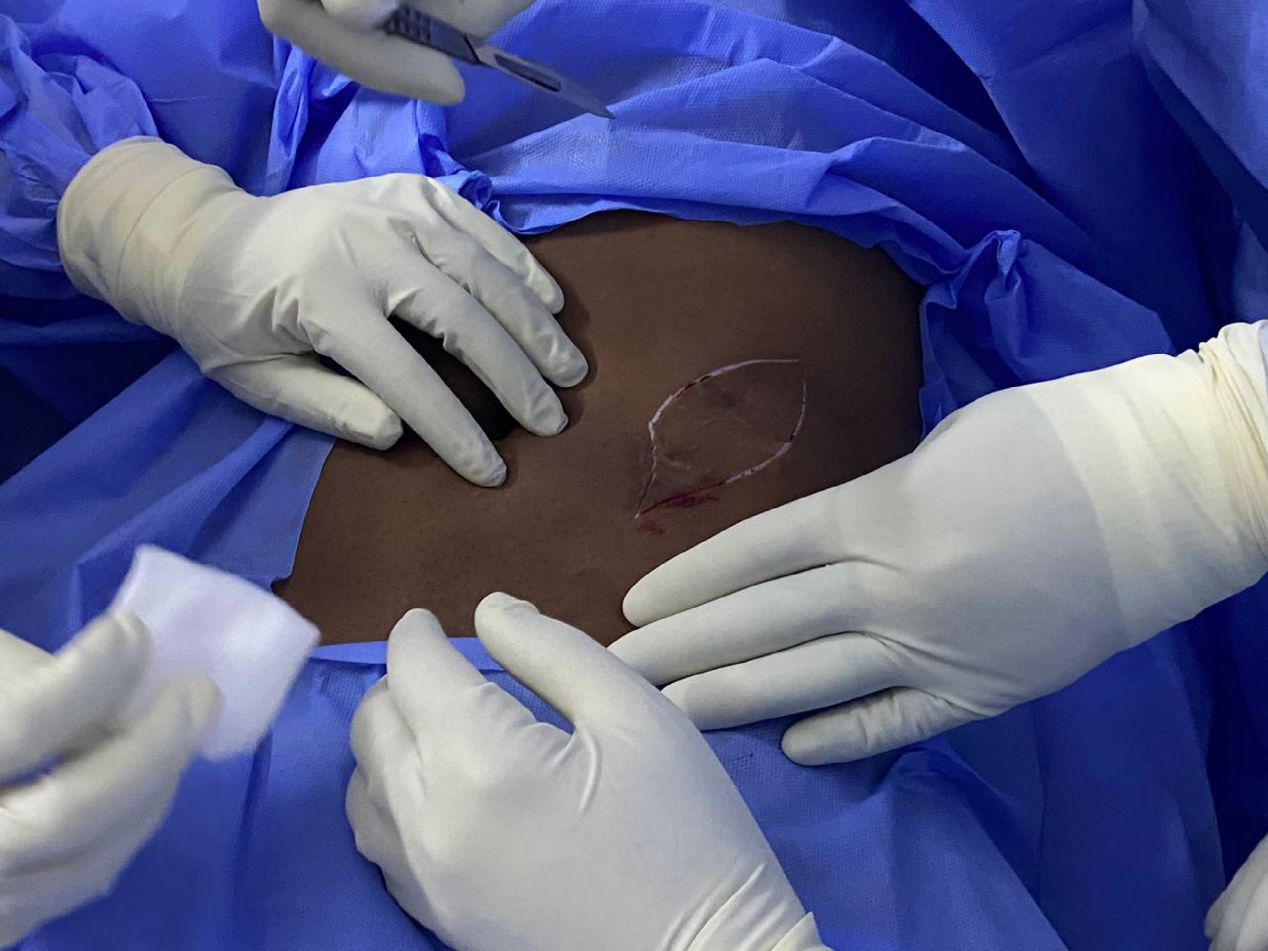
Illustrate a nontender, nonattached 4 × 3 cm mass palpable in the left lumbar region.

**FIGURE 2:**
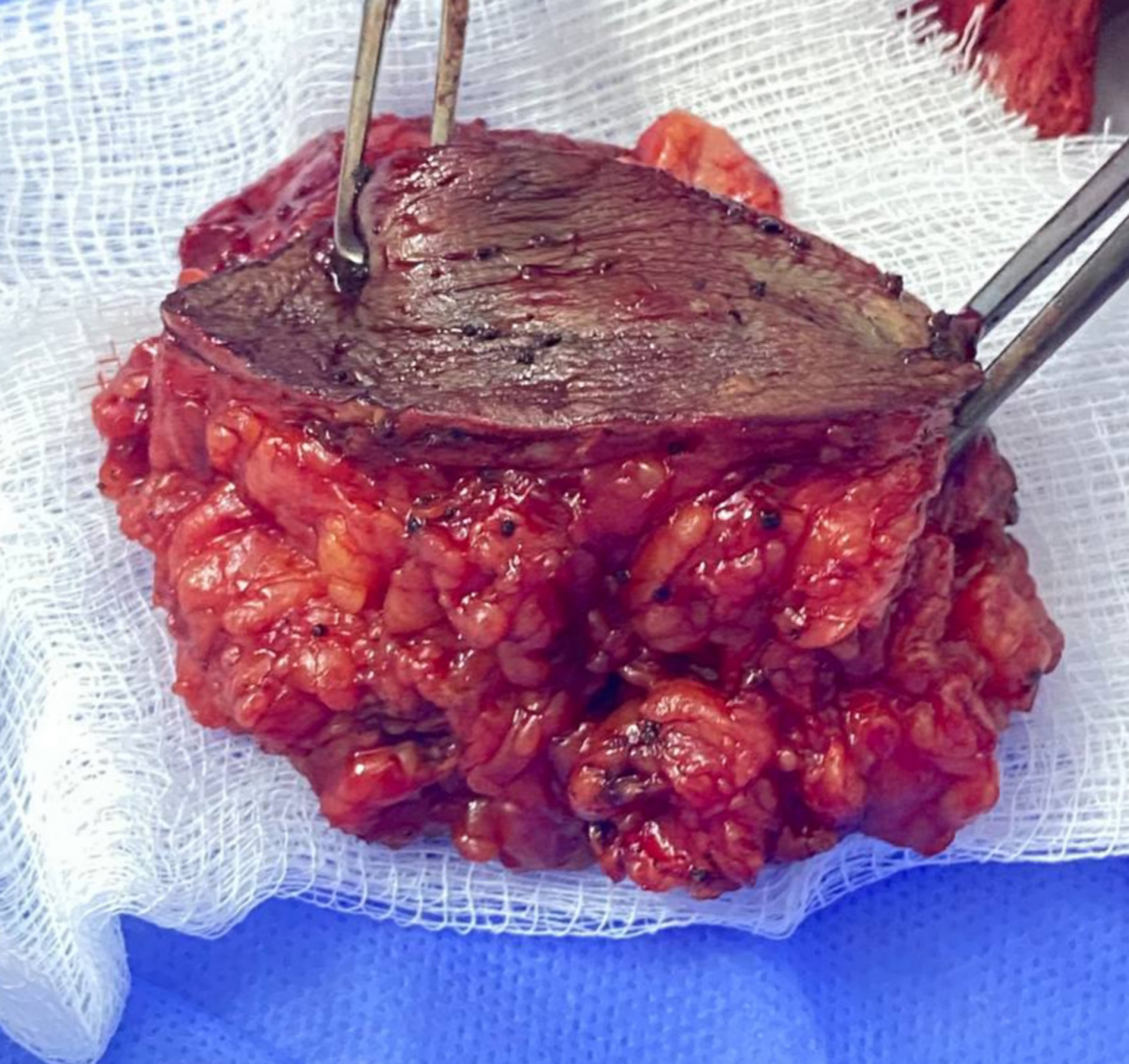
Illustrates the macroscopic appearance of the excised tissue, depicting the presence of black grains.

On the examination, the patient appeared well, with no signs of pallor or jaundice. His abdomen had a normal contour, was flat, and moved with respiration. Two surgery scars were observed, along with a small, visible mass in the left lumbar region (Figure 1). The hernia orifices were intact. A firm, nontender, nonattached 4 × 3 cm mass was palpable in the left lumbar region (Figure 1). An ultrasound of the anterior abdominal wall revealed a complete cystic mass measuring 1.8 × 1 cm, with solid echogenic content and mild fluid content. The patient underwent wide local excision (WLE) under general anesthesia, and the excised mass showed scattered black grains (Figure 2). A biopsy was obtained for histopathology analysis. For the histological investigation, the sample was stained with hematoxylin and eosin (H&E). The H&E section revealed multiple light brown grains surrounded by mixed inflammatory cells including neutrophils and multinucleated giant cells. This was suggestive for black grain eumycetoma with type I and II tissue reactions.

After the successful excision of the mass, the patient's treatment plan included an oral dosage of Itraconazole 200 mg, taken twice daily, for a minimum of 6 months.^1^ In addition, the healthcare provider requested monthly liver function tests to monitor treatment tolerability, considering the patient is living with a Hepatitis B infection. Interestingly, within 3 months of starting the treatment, the lesion has fully healed without any evidence of recurrence. To confirm this, an ultrasound examination was performed at the site, which showed no abnormalities. Additionally, the patient's liver remained unaffected, as no signs of liver damage were observed during this period.

## 3 DISCUSSION

This case presents an atypical presentation of eumycetoma affecting the abdominal wall. Eumycetoma is a rare and often overlooked diagnosis due to its varying clinical presentations.^2^, ^5^, ^6^ It is primarily caused by more than 70 species of fungi, with *Madurella* species being responsible for most cases reported worldwide.^2^ This diagnostic challenge has been exacerbated by the currently ongoing humanitarian crisis throughout the country that is characterized by the collapsed health system.^7^

Typically, eumycetoma presents with a classical triad of painless swelling, sinuses, and discharge‐containing grains.^2^ However, this case lacked the characteristic sinuses and grain discharge, posing a challenge in suspecting the condition. The diagnosis was made incidentally during surgical excision when embedded grains were discovered within the excised tissue, which was confirmed by histopathology analysis. This atypical presentation might be attributed to the preexisted hepatitis B infection and related immunological response. Unfortunately, the burden of hepatitis viruses is rapidly growing uncontrolled in the country despite the existence of effective prevention and control strategies globally.^8^, ^9^, ^10^, ^11^, ^12^

The patient in this case, a 28‐year‐old male from central Sudan, presented with a recurrent left‐sided abdominal wall mass that had gradually increased in size over 6 years. Despite two previous surgical excisions, the mass recurred each time. The most recent recurrence was smaller but still caused mild pain during heavy lifting. The full encapsulation of the grains led to the absence of the characteristic sinuses and discharge. This in turn, made the diagnosis of eumycetoma more challenging.

Regarding the management of eumycetoma, it is important to note that this condition is often refractory to medications, and a prolonged duration of treatment, typically around 6 months, is usually required. Currently, there are several available treatment options for eumycetoma.^13^, ^14^, ^15^ One common approach is the use of imidazoles such as ketoconazole (400 mg/day), itraconazole (200–400 mg/day), posaconazole (200 mg/day), and voriconazole (400–600 mg/day).^13^, ^14^, ^15^ Another option is the use of amphotericin B (0.5–1.25 mg/kg per day) or terbinafine (500–1000 mg/day).^16^, ^17^ These medications can be used alone or in combination, depending on the severity and specific characteristics of the infection.

In our reported patient, oral administration of itraconazole at a dosage of 200 mg twice daily was chosen due to its previous documented efficacy, availability, and relatively low cost compared to other treatment options. Additionally, surgical excision of the lesion was performed to enhance the effectiveness of the medical treatment.^18^ It is worth noting that even with treatment, relapses can occur, so regular follow‐up is necessary to ensure complete remission of the disease. Interestingly, The presence of hepatitis B in eumycetoma patients can indeed complicate the management of the condition. Treatments for eumycetoma, such as antifungal medications, may need to be adjusted or closely monitored in patients who also have hepatitis B. This is because antifungal affect the liver function,^19^ and close monitoring of liver function is necessary to ensure the safety and effectiveness of treatment.

Furthermore, hepatitis B infection weakens the immune system,^20^ making individuals more susceptible to infections, including fungal infections like eumycetoma. As a result, eumycetoma may be more severe or challenging to treat in patients with concurrent hepatitis B. It is crucial for healthcare providers to consider the patient's overall health, including the status of their hepatitis B infection, when developing a treatment plan. Particularly, in areas with high burden of hepatitis like Sudan.^8^, ^9^, ^10^, ^12^

Given these considerations, the presence of hepatitis B in eumycetoma patients requires careful management and monitoring by healthcare providers. A multidisciplinary approach may be necessary, involving infectious disease specialists, gastroenterologists, and dermatologists to ensure appropriate treatment and minimize the risk of complications.

Regular monitoring of liver function and proactive management of both conditions are essential to optimize treatment outcomes and minimize potential risks. It is essential for patients with both hepatitis B and eumycetoma to work closely with their healthcare providers to develop a tailored treatment plan that takes into account the individual's specific circumstances and medical history.

Furthermore, healthcare providers, especially in endemics areas, should be aware of uncommon presentations of NTDs and maintain a high suspicion for rare manifestations of diseases, particularly in limited resources and diagnostic capacity settings. Further research is needed to understand atypical presentations of eumycetoma infections and the associated risk factors to improve the early detection and case management.

In conclusion, this case highlights the importance of comprehensive thorough investigations and full documentation of health conditions. Recognizing and diagnosing atypical presentations are crucial for an effective case management and improving the clinical outcomes for patients. Healthcare providers should remain careful and consider rare diagnoses in their differential diagnosis, especially when dealing with chronic, recurrent lesions. Further studies are warranted to improve our understanding of the diverse clinical presentations of eumycetoma and identify potential diagnostic challenges. Improved awareness, early detection, and prompt interventions are essential to improve the public health of communities at risk and the quality of life for individuals affected by this neglected tropical disease, as well as prevent further complications.

## AUTHOR CONTRIBUTIONS

**Alaa Tajeldeen Habeeb Abdallah:** Conceptualization; data curation; formal analysis; investigation; methodology; supervision; validation; visualization; writing – review and editing. **Rami Elsiddig Abdelkhalig:** Conceptualization; data curation; formal analysis; funding acquisition; investigation; methodology; project administration; supervision; validation; visualization; writing – review and editing. **Elwasila Hamid:** Conceptualization; data curation; formal analysis; funding acquisition; investigation; methodology; project administration; resources; supervision; validation; visualization; writing – review and editing. **Ayman Ahmed:** Conceptualization; data curation; investigation; methodology; supervision; validation; visualization; writing – original draft; writing – review and editing. **Emmanuel Edwar Siddig:** Conceptualization; data curation; formal analysis; methodology; project administration; validation; visualization; writing – original draft; writing – review and editing.

## FUNDING INFORMATION

None.

## CONFLICT OF INTEREST STATEMENT

The author reports no conflicts of interest in this work.

## CONSENT

Written informed consent was obtained from the patient to publish this report in accordance with the journal's patient consent policy.

## Data Availability

The data that support the findings of this study are available from the corresponding author upon reasonable request.
